# Data on structural and composition-related merits of gC_3_N_4_ nanofibres doped and undoped with Au/Pd at the atomic level for efficient catalytic CO oxidation

**DOI:** 10.1016/j.dib.2019.104734

**Published:** 2019-10-30

**Authors:** Kamel Eid, Mostafa H. Sliem, Amal S. Eldesoky, Aboubakr M. Abdullah

**Affiliations:** a Center for Advanced Materials, Qatar University, Doha, P.O. Box 2713, Qatar; b Department of Biomedical Engineering, Higher Technological Institute, 10th of Ramadan, 228, Egypt

**Keywords:** CO oxidation, gC_3_N_4_, Greenhouse gases, One-dimensional, gC_3_N_4_ nanofibers

## Abstract

Precise design of graphitic carbon nitride (gC_3_N_4_) nanostructures is of grand importance in different catalytic applications. This article emphasizes additional data on the fabrication of metal-free gC_3_N_4_ nanofibres (gC_3_N_4_NFs) and its associated structural and composition analysis compared with Au/Pd co-doped gC_3_N_4_ nanofibres (Au/Pd/gC_3_N_4_NFs). The data is including the typical fabrication process of metal-free gC_3_N_4_ nanofibers and its SEM, TEM, and element mapping analysis beside Raman, and FTIR spectra relative to Au/Pd/gC_3_N_4_NFs. We also investigated the catalytic CO oxidation durability testes on Au/Pd/gC_3_N_4_NFs compared to Pd/gC_3_N_4_NFs and Au/gC_3_N_4_NFs. The presented data are associated with the research article entitled “Rational synthesis of one-dimensional carbon nitride-based nanofibers atomically doped with Au/Pd for efficient carbon monoxide oxidation.” [1].

**Table:** Specifications Table

Subject area	Chemistry
More specific subject area	Catalysis
Type of data	Scheme, Tables, Figures
How data was acquired	Transmission electron microscope ((TEM), TecnaiG220, FEI, Hillsboro, OR, USA) equipped with Energy Dispersive X-Ray Analysis (EDX), scanning electron microscope ((SEM), Hitachi S-4800, Hitachi, Tokyo, Japan), Raman spectroscopy (PerkinElmer Raman Station 400 spectrometer), and CO oxidation stability tests (online gas analyzer IR-200, Yokogawa, Japan).
Data format	The presented raw data are imaged and analyzed.
Experimental factors	The CO oxidation durability tests were carried out under continuous gas mixture gas flow while heating from room temperature to 300 °C.
Experimental features	The CO conversion durability tests were benchmarked as a function of temperature and metal dopants.
Data source location	Center for advanced materials, Qatar University, Doha P.O. Box 2713, Qatar.
Data accessibility	The data are available in this article
Related research article	Rational synthesis of one-dimensional carbon nitride-based nanofibers atomically doped with Au/Pd for efficient carbon monoxide oxidation.” [1]

**Table:** 

**Value of the Data** • The present data allowed controlling the shape and composition of gC_3_N_4_ nanofibers that paves the way for scientists to tailor and decipher the formation mechanism of gC_3_N_4_. • This data allowed understanding the architectural and compositional related merits of the gC_3_N_4_-based materials; thus, it is beneficent for controlling their properties for various catalytic applications. • Investigating the catalytic CO oxidation stability of Au/Pd/gC_3_N_4_NFs is essential for its scaling up for the commercial applications. • These data can serve as a benchmark for further development of new gC_3_N_4_-based nanostructures for CO conversion to CO_2_ and other gas conversion reactions.

## 1 Data

**Fig. 1:**
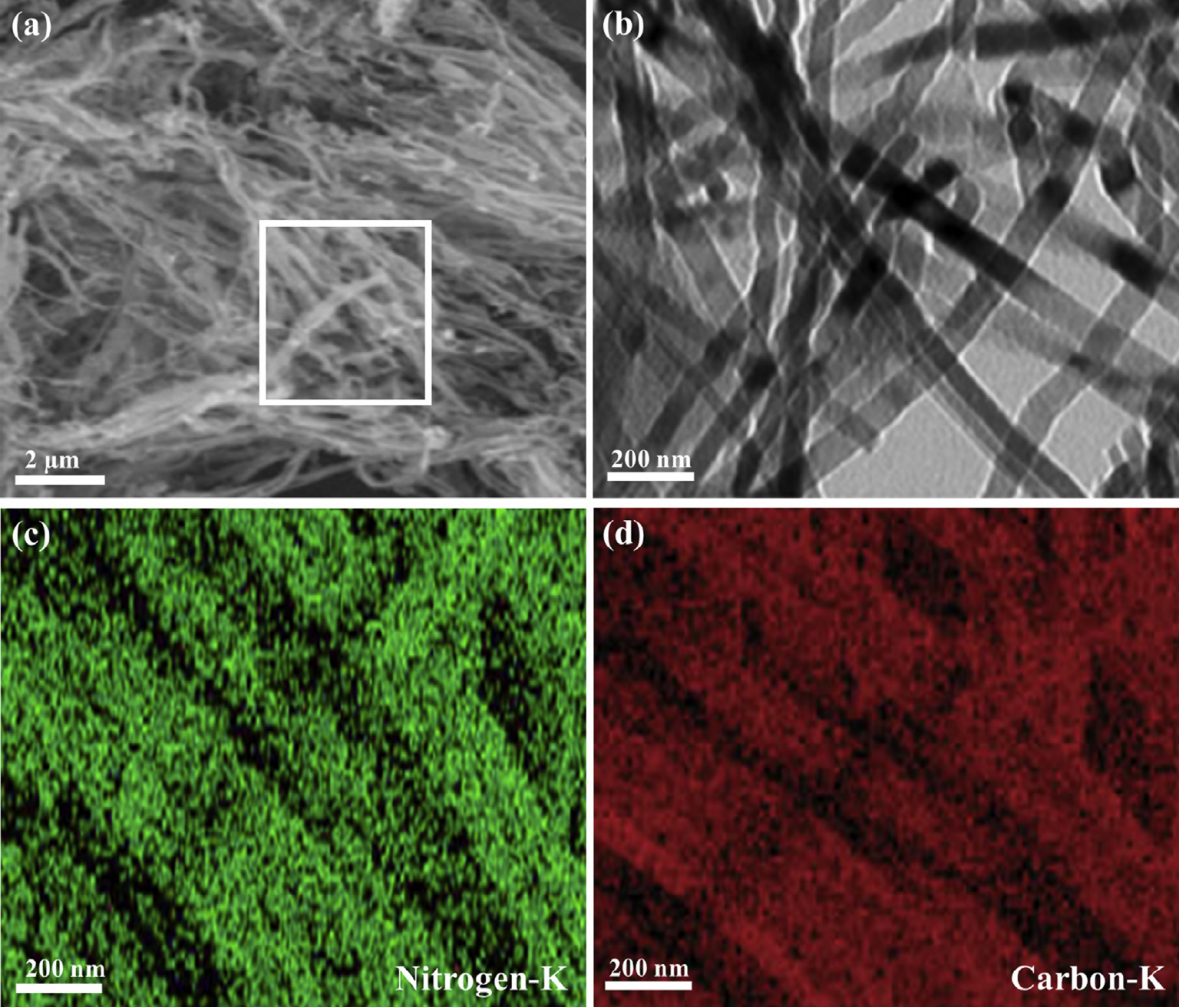
(a) SEM and (b) TEM images of typically synthesized gC_3_N_4_NFs. (c–d) Elemental mapping of nitrogen and carbon recorded from the marked area in (a). For the SEM and elemental mapping images, 2 mg of the powder was stacked on a carbon tab and imaged as it is. For the TEM analysis, 1 mg/mL of the powder was dispersed in ethanol solution, and 10 μl solution was mounted on a carbon-coated copper TEM grid and left to dry before the imaging.

**Fig. 2:**
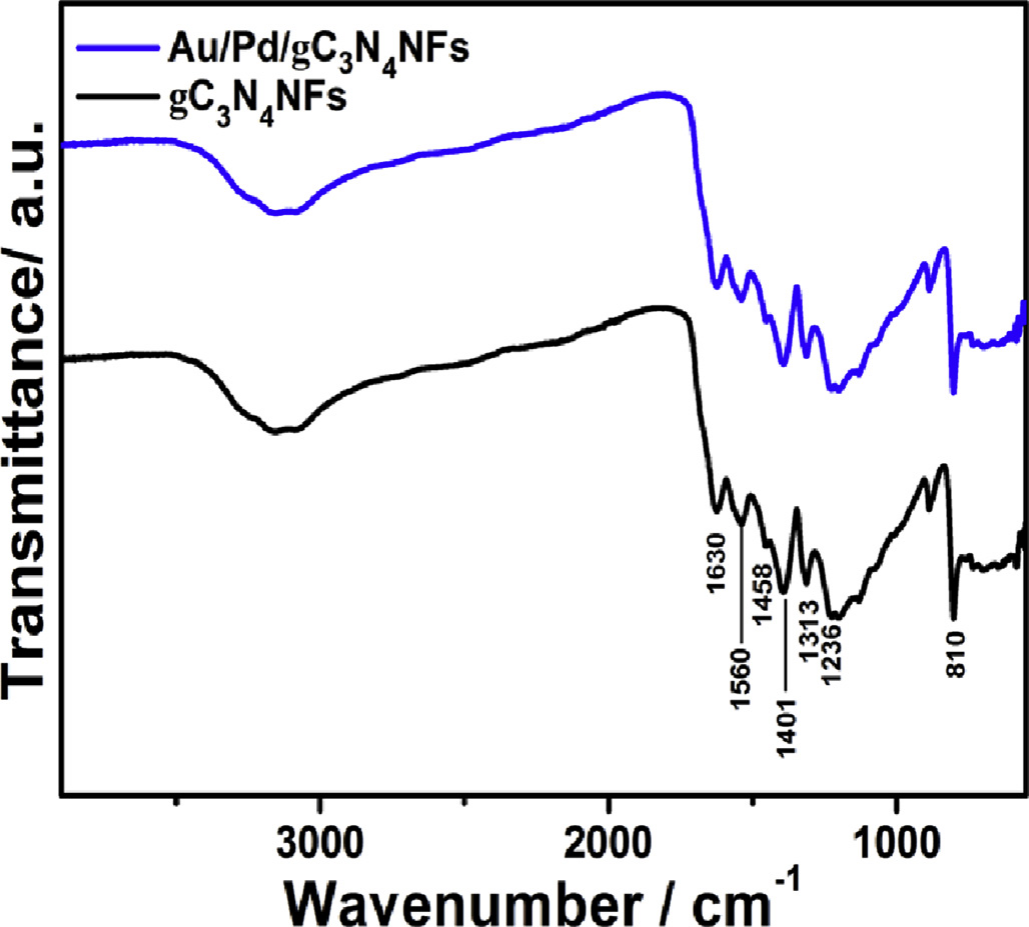
FTIR of the as-synthesized Au/Pd/gC_3_N_4_NFs and gC_3_N_4_NFs. Before the measurements, the samples were mixed with 0.1% of KBr powder followed by grinding for 3 min and then pressed into a pellet.

**Fig. 3:**
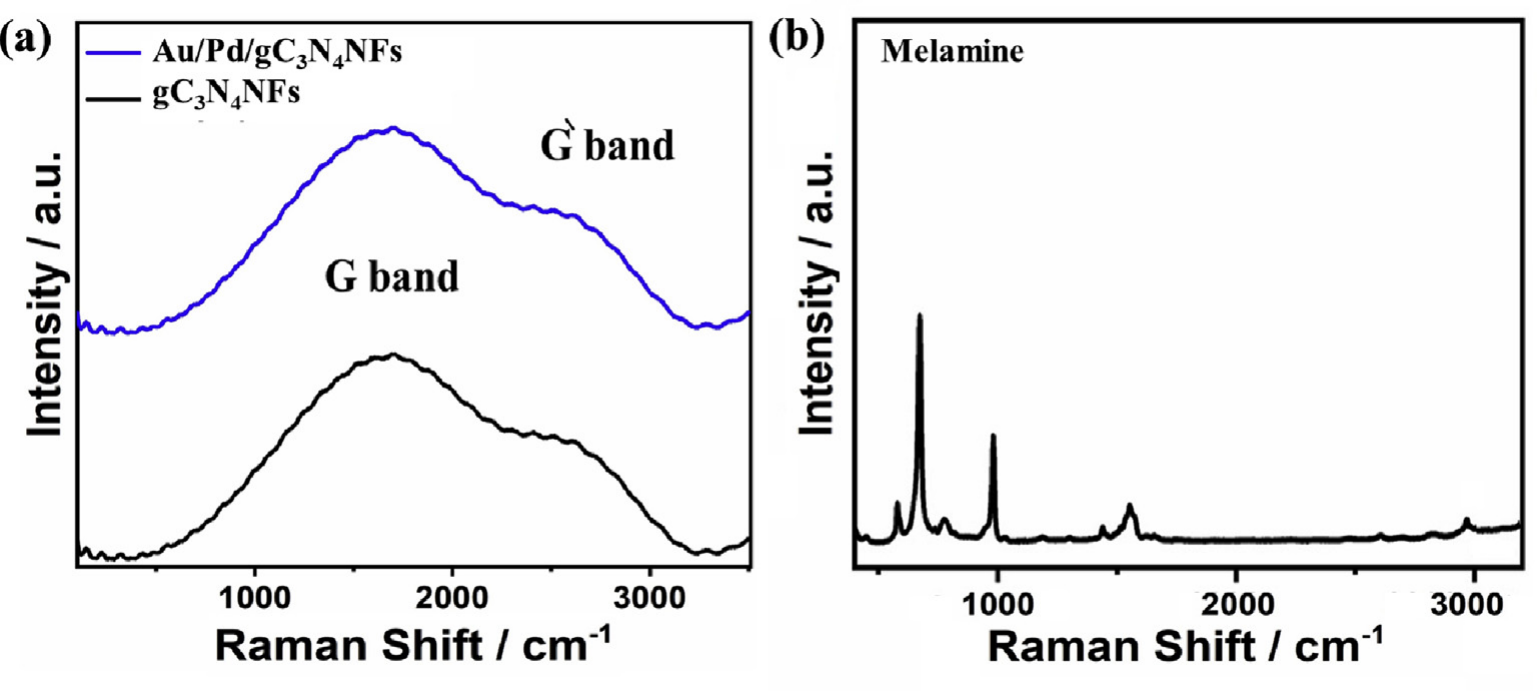
Raman spectra of (a) typically formed Au/Pd/gC_3_N_4_NFs and gC_3_N_4_NFs and (b) commercial melamine. The Raman spectra were recorded on a PerkinElmer Raman Station 400 spectrometer under 785 nm laser excitation. Before the measurements, the samples were dispersed in ethanol solution (2 wt %) and then deposited on a glass slide (0.5 × 0.5 cm^2^), and left to dry at room temperature.

**Fig. 4:**
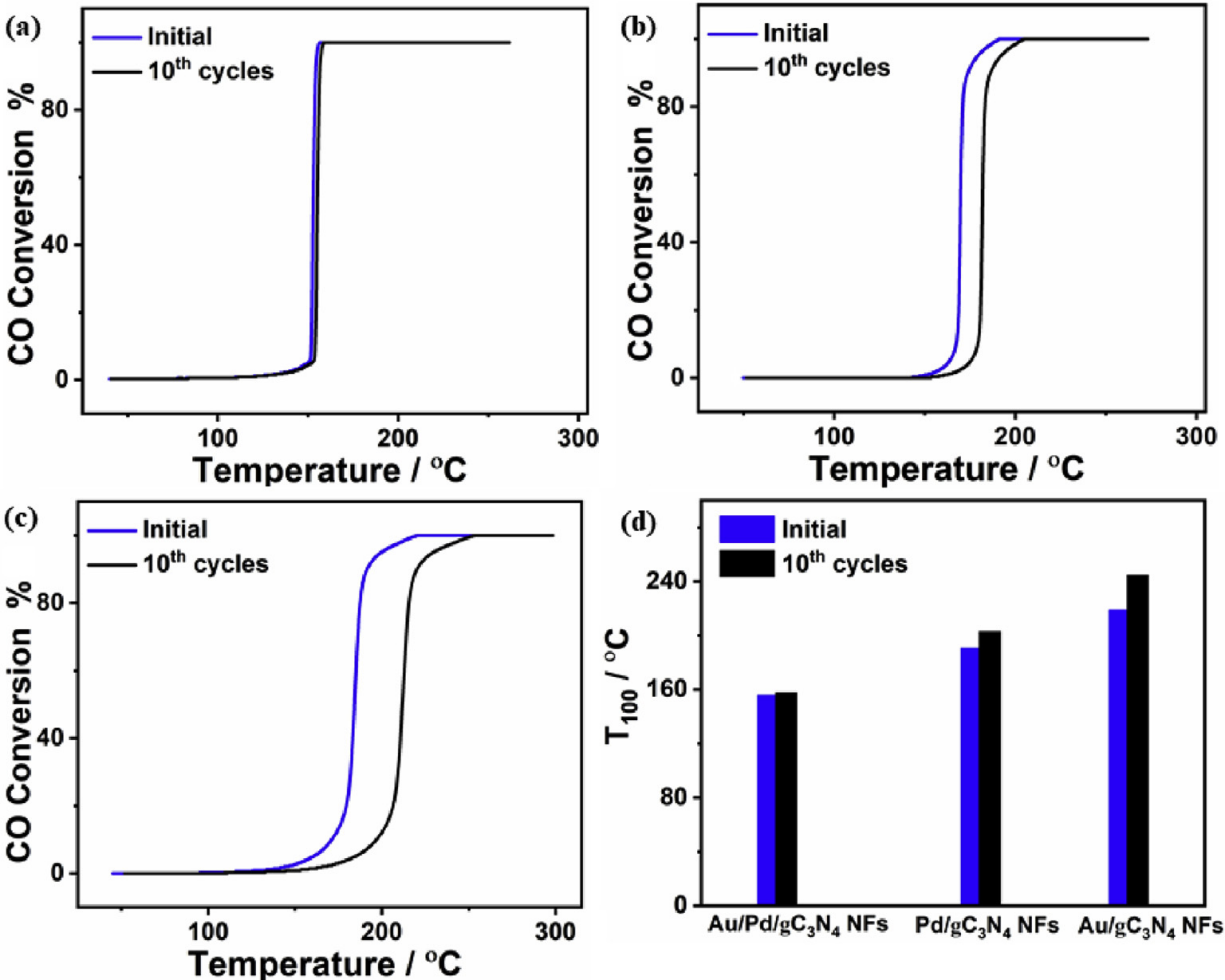
The CO oxidation durability tests over (a) Au/Pd/gC_3_N_4_NFs, (b) Pd/gC_3_N_4_NFs, and (c) Au/gC_3_N_4_NFs. (d) Comparison between the T_100_ before and after the stability tests on the as-synthesized catalysts.

**Fig. 5:**
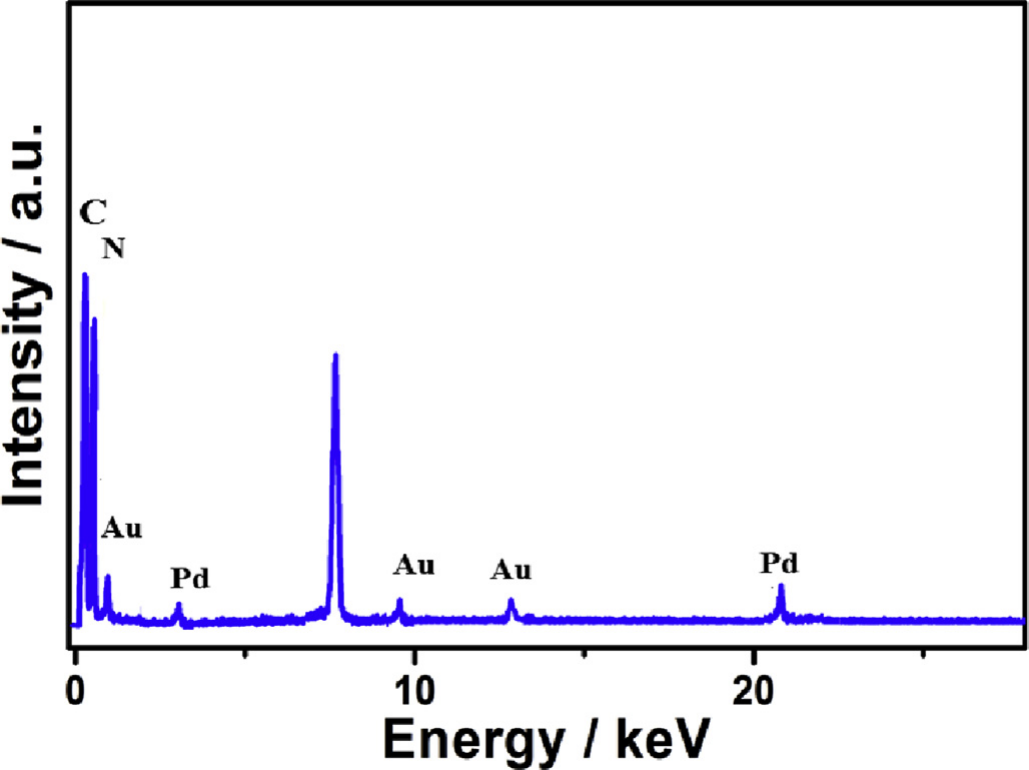
The EDX analysis of Au/Pd/gC_3_N_4_NFs after the CO oxidation durability tests.

**Fig. 6:**
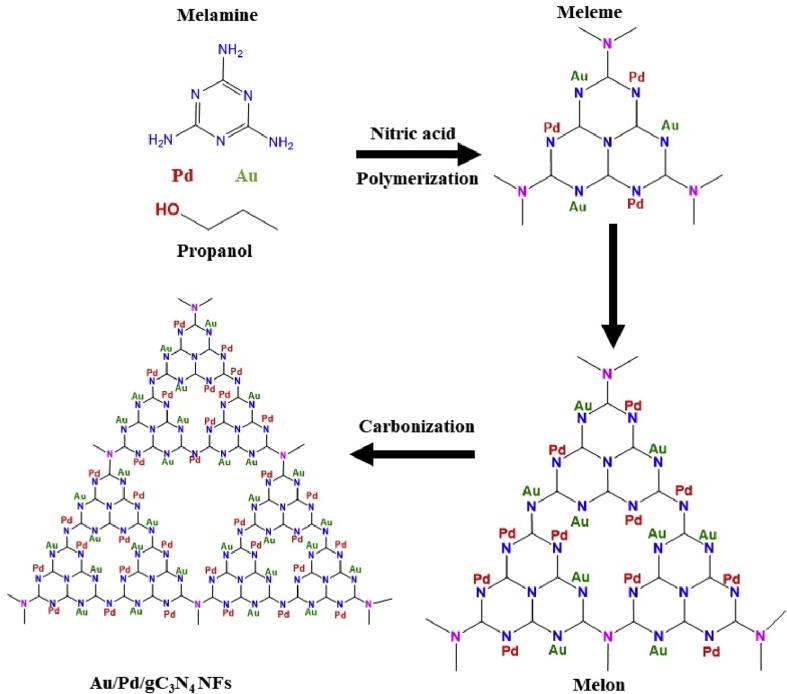
A scheme illustrates the synthetic mechanism process of Au/Pd/gC_3_N_4_ nanofibers and the distribution of both Au and Pd inside the skeletal structure of gC_3_N_4_.

The presented herein data provides deep insights on the rational synthesis of metal-free gC_3_N_4_NFs and its correlated analysis relative to Au/Pd/gC_3_N_4_NFs. This is in addition to the CO oxidation durability of Au/Pd/gC_3_N_4_NFs and its compositional analysis after CO oxidation reaction. Particularly, the data involves the SEM, TEM, and elemental mapping images of gC_3_N_4_NFs (Fig. 1), while the FTIR and Raman spectra of gC_3_N_4_NFs compared to Au/Pd/gC_3_N_4_ are represented in Fig. 2 and Fig. 3, respectively. Meanwhile, the CO oxidation stability testes carried out on Au/Pd/gC_3_N_4_NFs, Pd/gC_3_N_4_NFs, and Au/gC_3_N_4_NFs beside their loss in the complete conversion temperature (T_100_) are shown in (Fig. 4). This is alongside the Energy Dispersive X-ray Analysis (EDX) analysis of Au/Pd/gC_3_N_4_NFs after the CO oxidation durability testes (Fig. 5) and the schematic reveals the synthetic mechanism process of Au/Pd/gC_3_N_4_NFs in (Fig. 6).

## 2 Experimental design, materials, and methods

### 2.1 Synthesis of metal-free gC_3_N_4_NFs

Fig. 1 shows the SEM and TEM images of metal-free gC_3_N_4_NFs typically synthesized by the slow dispersion of melamine (1 g) in an aqueous solution of isopropanol (30 mL, 99%) under stirring at 40 °C. Then, an aqueous solution of nitric acid (HNO_3_, 60 mL, 0.3 M) was added to the previous solution under stirring at 40 °C. The as-formed white precipitate was filtered and washed with isopropanol solution before being dried at 100 °C for 12 h. Finally, the obtained powder was subsequently annealed under nitrogen at 550 °C for 2 h (5 °C min^−1^).

The fabrication of Au/Pd/gC3N4NFs was done according to the same procedure of metal-free gC3N4NFs but in presence of Au and Pd precursors before the addtion of HNO_3_ (see Ref. [1] for more information).

The SEM image clearly shows the formation of uniform one-dimensional fiber-like morphology in high yield (nearly 100%) without resolving any other undesired shapes such as spherical and sheets (Fig. 1a). The average length of thus formed nanofibers obtained from the TEM is about 10 μm. The TEM image further confirmed the formation of a nanofiber structure with smooth surfaces and had an average width of nearly 80 ± 2 nm (Fig. 1b). The element mapping analysis indicated the presence of both C and N with an atomic ratio of 41 and 59, respectively (Fig. 1c and d).

### 2.2 Chemical structure and composition analysis

The chemical bonds and the functional groups of both Au/Pd/gC_3_N_4_NFs and gC_3_N_4_NFs were evaluated using the Fourier transform infrared (FTIR) analysis. Both Au/Pd/gC_3_N_4_NFs and gC_3_N_4_NFs revealed the peaks attributed to the stretching vibration of triazine at 810 cm^−1^ and several peaks for C–N heterocycles from 1000 to 1750 cm^−1^ (Fig. 2) [2]. The weak bands observed between 2900 and 3300 cm^−1^ are assigned to the N–H vibrations at the edges of gC_3_N_4_-based material. The anchoring of Au and Pd over N-atoms inside Au/Pd/gC_3_N_4_NFs slightly broadens and decreases in the intensity of N–H and C–N bands of Au/Pd/gC_3_N_4_NFs [[1], [2], [3], [4]].

Fig. 3a shows the Raman spectra of gC_3_N_4_NFs, compared to Au/Pd/gC_3_N_4_NFs. Both materials revealed a sharp peak at 1555 cm^−1^ of graphitic (G) band, which indicates the high degree of graphitization of the as-obtained materials [4,5]. The G band of Au/Pd/gC_3_N_4_NFs was slightly positively shifted relative to that of gC_3_N_4_NFs, implying its higher strained effect. Additionally, both materials displayed a small spectrum at 2690 cm^−1^ of (Gˋ peak), resulting from the disordered surface. Fig. 3b shows the typical spectrum of melamine starting from 500 until 3000 cm^−1^, which are dissimilar to those recorded for Au/Pd/gC_3_N_4_NFs and gC_3_N_4_NFs [[1], [2], [3]]. Table 1 summarizes the identification and position for Raman spectra of Au/Pd/gC_3_N_4_NFs and gC_3_N_4_NFs.

**Table 1:** The position of the resolved Raman spectra of the as-prepared materials.

Materials	G-band	D-band	(Gˋ-band)
Au/Pd/gC_3_N_4_NFs	1555 cm^−1^	1360 cm^−1^	2690 cm^−1^
gC_3_N_4_NFs	1554 cm^−1^	1359 cm^−1^	2689 cm^−1^

### 2.3 CO oxidation stability tests

The CO oxidation is of particular interest in wide varieties of industrial, biological, and environmental remediation applications [2,[6], [7], [8]]. Thus, it is essential to develop efficient and durable catalysts for CO oxidation reaction to convert highly toxic CO gas into less toxic gasses or other fuels [[1], [2], [3], [4],[8], [9], [10], [11]]. After determination, the complete CO conversion temperature (T_100_) on the as-synthesized Au/Pd/gC_3_N_4_NFs, Pd/gC_3_N_4_NFs, and Au/gC_3_N_4_NFs, the long-term durability tests were investigated at their T_100_ for 48 h. In particular, the catalysts were exposed to the gas mixture consisting of CO (4%), O_2_ (20%), and Ar (76%) with a total flow of 50 mL min^−1^ and the temperature was increased steadily (5 °C min^−1^) until the T_100_ of each catalyst. Then, the percentage of CO conversion was monitored through an online multichannel infrared gas analyzer (IR200, Yokogawa, Japan). Following the durability tests, the CO conversion efficiencies were measured again through the pretreatment at 250 °C under an O_2_ flow of 50 mL min^−1^, and H_2_ (30 mL min^−1^) for 1 h. Then, each catalyst was exposed to a gas mixture of CO (4%), O_2_ (20%), and Ar (76%) with a total flow of 50 mL min^−1^, while heating from the room temperature till the complete CO conversion occurred.

Fig. 4 shows the CO oxidation durability of Au/Pd/gC_3_N_4_NFs compared to Pd/gC_3_N_4_NFs, and Au/gC_3_N_4_NFs. In particular, after the accelerated durability tests, Au/Pd/gC_3_N_4_NFs reserved its initial CO oxidation activity without any noticed loss (Fig. 4a); meanwhile, Pd/gC_3_N_4_NFs loss is around 7% (Fig. 4b) and Au/gC_3_N_4_NFs lose about 11% (Fig. 4c). However, from the light-off curves for the CO conversion durability expressed as a function of time, all materials did not show any noticed change in the CO oxidation kinetics. To this end, the estimated T_100_ after the stability cycles on Au/Pd/gC_3_N_4_NFs, Pd/gC_3_N_4_NFs, and Au/Pd/gC_3_N_4_NFs were about 146 °C, 203 °C, and 246.4 °C, respectively (Fig. 4d).

### 2.4 Compositional stability

After the CO oxidation durability tests, the elemental composition of Au/Pd/gC_3_N_4_NFs was carried out using the EDX analysis to examine any changes in the composition. Fig. 5 shows the EDX analysis of Au/Pd/gC_3_N_4_NFs, which revealed the presence of C, N, Au, and Pd without any changes or undesired phases. The detailed atomic ratios of C/N/Au/Pd are about with 39/60/0.51/0.44, respectively.

Chemically speaking, and looking deeply to the formation mechanism, Au/Pd/gC_3_N_4_NFs combine between the unique physicochemical properties of gC_3_N_4_ and catalytic merts of Au/Pd atomic dopants [[1], [2], [3], [4],[12], [13], [14], [15]]. Particularly, the strong binding affinity between N-atoms of melamine and metal atoms Au/Pd led to their chemical bonding in the form of -N-Au and -N-Pd during the polymerization step resulting in a coherent distribution through the skeletal structure of gC_3_N_4_NFs (Fig. 6) [[1], [2], [3], [4]]. These chemical legends not only allow the homogenous distribution of Au/Pd inside the nanofibers but also stabilize them against the detachment and agglomeration, during the CO oxidation reaction.

## References

[1] Eid K., Sliem M.H., Eldesoky A.S., Al-Kandari H., Abdullah A.M. (2019). Rational synthesis of one-dimensional carbon nitride-based nanofibers atomically doped with Au/Pd for efficient carbon monoxide oxidation. Int. J. Hydrogen Energy.

[2] Eid K., Abdullah A.M. (2019). Data on the catalytic CO oxidation and CO_2_ reduction durability on gC_3_N_4_ nanotubes Co-doped atomically with Pd and Cu. Data in Brief.

[3] Eid K., Sliem M.H., Al-Kandari H., Sharaf M.A., Abdullah A.M. (2019). Rational synthesis of porous graphitic-like carbon nitride nanotubes codoped with Au and Pd as an efficient catalyst for carbon monoxide oxidation. Langmuir.

[4] Fu Y., Zhu J., Hu C., Wu X., Wang X. (2014). Covalently coupled hybrid of graphitic carbon nitride with reduced graphene oxide as a superior performance lithium-ion battery anode. Nanoscale.

[5] Han C., Gao Y., Liu S., Ge L., Xiao N., Dai D., Xu B., Chen C. (2017). Facile synthesis of AuPd/g-C_3_N_4_ nanocomposite: an effective strategy to enhance photocatalytic hydrogen evolution activity. Int. J. Hydrogen Energy.

[6] Dehghani M.H., Jarahzadeh S., Hadei M., Mansouri N., Rashidi Y., Yousefi M. (2018). The data on the dispersion modeling of traffic-related PM_10_ and CO emissions using CALINE3; A case study in Tehran, Iran. Data in Brief.

[7] Anawe P.A.L., Adewale F.J. (2018). Data on physico-chemical, performance, combustion and emission characteristics of Persea Americana Biodiesel and its blends on direct-injection, compression-ignition engines. Data in Brief.

[8] Eid K., Sliem M.H., Abdullah A.M. (2019). Unraveling template-free fabrication of carbon nitride nanorods codoped with Pt and Pd for efficient electrochemical and photoelectrochemical carbon monoxide oxidation at room temperature. Nanoscale.

[9] Ward T., Delannoy L., Hahn R., Kendell S., Pursell C.J., Louis C., Chandler B.D. (2013). Effects of Pd on catalysis by Au: CO adsorption, CO oxidation, and cyclohexene hydrogenation by supported Au and Pd-Au catalysts. ACS Catal..

[10] Chen H.-L., Su C.-H., Chen H.-T. (2012). Catalytic CO oxidation by Au–Pd core-shell nanoparticles: a first-principles study. Chem. Phys. Lett..

[11] Xu J., White T., Li P., He C., Yu J., Yuan W., Han Y. (2010). Biphasic Pd−Au alloy catalyst for low-temperature CO oxidation. J. Am. Chem. Soc..

[12] Tahir M., Cao C., Mahmood N., Butt F.K., Mahmood A., Idrees F., Hussain S., Tanveer M., Ali Z., Aslam I. (2014). Multifunctional g-C_3_N_4_ nanofibers: a template-free fabrication and enhanced optical, electrochemical, and photocatalyst properties. ACS Appl. Mater. Interfaces.

[13] Gao J., Zhou Y., Li Z., Yan S., Wang N., Zou Z. (2012). High-yield synthesis of millimetre-long, semiconducting carbon nitride nanotubes with intense photoluminescence emission and reproducible photoconductivity. Nanoscale.

[14] Sun S., Liang S. (2017). Recent advances in functional mesoporous graphitic carbon nitride (mpg-C_3_N_4_) polymers. Nanoscale.

[15] Zhao Z., Sun Y., Dong F. (2015). Graphitic carbon nitride based nanocomposites: a review. Nanoscale.

